# Hemorrhage enhances cytokine, complement component 3, and caspase-3, and regulates microRNAs associated with intestinal damage after whole-body gamma-irradiation in combined injury

**DOI:** 10.1371/journal.pone.0184393

**Published:** 2017-09-21

**Authors:** Juliann G. Kiang, Joan T. Smith, Marsha N. Anderson, Thomas B. Elliott, Paridhi Gupta, Nagaraja S. Balakathiresan, Radha K. Maheshwari, Barbara Knollmann-Ritschel

**Affiliations:** 1 Radiation Combined Injury Program, Armed Forces Radiobiology Research Institute, Bethesda, Maryland, United States of America; 2 Department of Pharmacology and Molecular Therapeutics, Uniformed Services University of the Health Sciences, Bethesda, Maryland, United States of America; 3 Department of Medicine, Uniformed Services University of the Health Sciences, Bethesda, Maryland, United States of America; 4 Department of Pathology, Uniformed Services University of the Health Sciences, Bethesda, Maryland, United States of America; University of California Davis, UNITED STATES

## Abstract

Hemorrhage following whole-body γ-irradiation in a combined injury (CI) model increases mortality compared to whole-body γ-irradiation alone (RI). The decreased survival in CI is accompanied by increased bone marrow injury, decreased hematocrit, and alterations of miRNA in the kidney. In this study, our aim was to examine cytokine homeostasis, susceptibility to systemic bacterial infection, and intestinal injury. More specifically, we evaluated the interleukin-6 (IL-6)-induced stress proteins including C-reactive protein (CRP), complement 3 (C3), Flt-3 ligand, and corticosterone. CD2F1 male mice received 8.75 Gy ^60^Co gamma photons (0.6 Gy/min, bilateral) which was followed by a hemorrhage of 20% of the blood volume. In serum, RI caused an increase of IL-1, IL-2, IL-3, IL-5, IL-6, IL-12, IL-13, IL-15, IL-17A, IL-18, G-CSF, CM-CSF, eotaxin, IFN-γ, MCP-1, MIP, RANTES, and TNF-α, which were all increased by hemorrhage alone, except IL-9, IL-17A, and MCP-1. Nevertheless, CI further elevated RI-induced increases of these cytokines except for G-CSF, IFN- γ and RANTES in serum. In the ileum, hemorrhage in the CI model significantly enhanced RI-induced IL-1β, IL-3, IL-6, IL-10, IL-12p70, IL-13, IL-18, and TNF-α concentrations. In addition, *Proteus mirabilis* Gram(-) was found in only 1 of 6 surviving RI mice on Day 15, whereas *Streptococcus sanguinis* Gram(+) and *Sphingomonas paucimobilis* Gram(-) were detected in 2 of 3 surviving CI mice (with 3 CI mice diseased due to inflammation and infection before day 15) at the same time point. Hemorrhage in the CI model enhanced the RI-induced increases in C3 and decreases in CRP concentrations. However, hemorrhage alone did not alter the basal levels, but hemorrhage in the CI model displayed similar increases in Flt-3 ligand levels as RI did. Hemorrhage alone altered the basal levels of corticosterone early after injury, which then returned to the baseline, but in RI mice and CI mice the increased corticosterone concentration remained elevated throughout the 15 day study. CI increased 8 miRNAs and decreased 10 miRNAs in serum, and increased 16 miRNA and decreased 6 miRNAs in ileum tissue. Among the altered miRNAs, CI increased miR-34 in the serum and ileum which targeted an increased phosphorylation of ERK, p38, and increased NF-κB, thereby leading to increased iNOS expression and activation of caspase-3 in the ileum. Further, let-7g/miR-98 targeted the increased phosphorylation of STAT3 in the ileum, which is known to bind to the iNOS gene. These changes may correlate with cell death in the ileum of CI mice. The histopathology displayed blunted villi and villus edema in RI and CI mice. Based on the *in silico* analysis, miR-15, miR-99, and miR-100 were predicted to regulate IL-6 and TNF. These results suggest that CI-induced alterations of cytokines/chemokines, CRP, and C3 cause a homeostatic imbalance and may contribute to the pathophysiology of the gastrointestinal injury. Inhibitory intervention in these responses may prove therapeutic for CI and improve recovery of the ileal morphologic damage.

## Introduction

Many victims suffered from radiation injury (RI) at Hiroshima and Nagasaki, Japan, in 1945. Among the victims, 60% received RI alone and approximately 40% of had other concurrent injuries in addition to the radiation injury [1, 2]. The RI combined with another injury, such as skin burn, wound, or hemorrhage (Hemo), is described as combined injury (CI). After the Chernobyl, reactor meltdown in 1986 in Ukraine, 10% of 237 victims exposed to RI received thermal burns [3]. In *in vivo* experiments, using mice [4–18], rats [19, 20], guinea pigs [21], dogs [22], and swine [23, 24], skin burns, wounds, or Hemo usually caused increased mortality after an otherwise non-lethal irradiation.

Ionizing radiation perturbs hematopoiesis in the bone marrow, which, in turn, decreases production of peripheral blood cells [17, 18, 25, 26]. RI breaks down the gastrointestinal (GI) barrier [27] and causes systemic bacterial infection, that is, sepsis [8], depresses the innate immune responses against infectious agents, including production of immunoglobulins, and disturbs the inflammatory responses, including C-reactive protein (CRP), complement component 3 (C3), [10] and the normal balance of inflammatory and anti-inflammatory cytokines and chemokines [8]. CRP is produced by the liver and is a biomarker for general stress response, whose production is a general response to inflammation or infectious agents [28]. A rise in concentrations of IL-6 in serum, which is produced predominantly by macrophages [29] and adipocytes [30], leads to increases in CRP [31].

It is evident that RI combined with wounds, burns, or Hemo augments the RI-induced acute radiation syndrome (ARS) [8, 10, 17]. But it was not clear whether Hemo in the CI model would amplify the RI-induced changes in CRP and C3 in blood and sepsis, like the observations resulted from RI combined with a wound or burn, i.e. in CI [10]. Therefore, it is imperative to measure CRP and C3 in this animal model of radiation combined with Hemo.

γ-Irradiation alters corticosterone concentrations in blood [32]. Corticosterone is an adrenal corticosteroid hormone that contributes to regulation of immune and stress responses in rodents. The dynamics of corticosterone depend on the intensity of irradiation and the time after irradiation [32]. Therefore, it is of interest to determine any alterations in the blood corticosterone concentrations after irradiation followed by hemorrhage.

MicroRNAs (miRNAs) are small, endogenous noncoding RNAs that regulate post transcriptional gene expression. MiRNAs have been shown to regulate various biological processes involved in kidney diseases such as hypoxia, differentiation, inflammation, cell proliferation, cell death, and fibrosis [33]. We have found that RI combined with Hemo (i.e. CI) regulates several miRNAs in kidney [17]. However, the effect of CI on miRNA expression pattern in the blood and ileum of mice and their role in inflammation, intestinal morphology, and sepsis are still unknown. In this study we investigated the CI-specific pathophysiology which may by induced by miRNA modulation. Understanding whether the synergistic effects of radiation and hemorrhage on induction of inflammation and sepsis are present following CI is imperative to determining the preventative measures that can be utilized to save lives in the event of nuclear disasters.

The aim of this study was to determine whether non-lethal Hemo, when following sub-lethal RI (i.e., combined injury, CI), exacerbated the effects of radiation exposure on inflammation, intestinal integrity, and sepsis. Response to Hemo alone and RI alone were shown to be regulated by NF-κB/iNOS [8] and miRNAs [17]. Therefore, we hypothesized that CI would result in an enhanced inflammatory response in the intestine compared to either Hemo alone or RI alone, which might be associated with NF-κB/iNOS and miRNAs. Mice were used to test our hypothesis because the intricate complex interactions among organs, tissues, and relevant molecular components of transcription factors, cytokines, and miRNA require a whole animal model. Our data support the conclusion that the detected alterations may contribute to intestinal deterioration caused by CI and could elucidate effective therapeutic interventions for treating CI.

## Materials and methods

### Ethics statement

Research was conducted in a facility accredited by the Association for Assessment and Accreditation of Laboratory Animal Care-International (AAALACI). All procedures involving animals were reviewed and approved by the Armed Forces Radiobiology Research Institute (AFRRI) Institutional Animal Care and Use Committee (IACUC). Euthanasia was carried out in accordance with the recommendations and guidelines of the American Veterinary Medical Association. For the survival study, we observed animals every 2 hours during work hours, and moribund animals were euthanized according to humane endpoints. The clinical definition of moribund is the state of dying with no expectation of recovery, where animals display a combination of the following: lowered body temperature, slow or impaired motion, continuous shaking, hunched back, and inability to maintain sternal recumbency. Moribund animals were euthanized in a separate cage into which carbon dioxide gas was applied until no breathing was observed in the mice, followed by a cervical dislocation as a secondary confirmatory method of euthanasia. Deceased animals were promptly removed from the cages to avoid any deterioration of tissues among surviving mice and preserve the integrity of the experiment. Any surviving animals at the end of the study were also euthanized by inhalation of carbon dioxide followed by cervical dislocation. For the studies other than those testing survival, mice at specific endpoints were anesthetized by isoflurane inhalation for the entire period of blood collection by cardiac puncture, immediately followed by a confirmatory cervical dislocation for euthanasia and terminal tissue collection.

### Animals and experimental design

Male CD2F1 mice (10 weeks old), were obtained from Harlan Laboratories, Inc. (Indianapolis, IN) and allowed to acclimate to their surroundings for 14 days prior to initiation of the study. All animals were randomly group housed in a room in which temperature (68–75°F), relative humidity (50±20%), and light (12-hr light-dark cycle) were controlled. The mice were randomly divided into four experimental groups (N = 6/group for mechanistic elucidation): Sham irradiation (0 Gy), Hemo (acute removal of 20% total blood volume), RI, or RI+Hemo (= CI). After injuries, mice were assigned to clean cages with 2–4 mice/cage and provided with proper food (standard rodent chow, Harlan Teklad 8604) and steam-sterilized, acidified water *ad libitum*. The health status of animals was monitored daily.

### Radiation injury (RI)

Mice were placed in well-ventilated acrylic restrainers and one whole-body dose of 8.75 Gy ^60^Co γ-photon radiation [17] was delivered at a dose rate of approximately 0.6 Gy/min. Dosimetry was performed using the alanine/electron paramagnetic resonance system. Calibration of the dose rate with alanine was traceable to the National Institute of Standards and Technology and the National Physics Laboratory of the United Kingdom. Sham-irradiated mice were placed in the same acrylic restrainers, taken to the radiation facility, and restrained for the same time as required for actual irradiation.

### Hemorrhage (Hemo) procedure

Within 2 hours post-RI, mice were anesthetized under isoflurane (~3%) and bled 0% (Sham or RI) or 20% (Hemo or RI+Hemo) of total blood volume via the submandibular vein as previously described [17, 34, 35]. Briefly, the jaw of an anesthetized mouse was cleaned with a 70% ethanol wipe, and glycerol was applied to the surface of the jaw to allow for ease of collection and measurement of blood loss. A 5-mm Goldenrod animal lancet (MEDIpoint, Inc, Mineola, NY) was used to puncture the submandibular vein of the mouse to collect facial-vein blood samples. A 75-mm heparinized hematocrit collection tube (Drummond Scientific Co., Broomall, PA) was marked and used to collect the appropriate amount of blood to ensure that 20% of total blood volume was extracted during the hemorrhage procedure. The volume of blood collected was based upon each individual mouse’s body mass [17, 34].

### Blood collection

Whole blood was collected by terminal cardiac puncture from mice (N = 6/group per time point) anesthetized by isoflurane (~3%) at several time points after sham, Hemo, RI or CI treatments. CapiJect tubes (Terumo, Somerset, NJ) were used to separate sera by centrifugation at 3,500 g for 90 sec. Sera were stored at −70°C until assayed (N = 6 per group) in biochemical assays, including cytokines/chemokines, corticosterone, CRP, C3, and miRNA.

### Cytokine and chemokine measurements

Cytokine concentrations were analyzed using the Bio-Plex Pro^™^ Mouse Cytokine Panel Plex (Bio-Rad; Hercules, CA) following the manufacturer’s directions. Briefly, serum from each animal was diluted fourfold and examined in duplicate. Data were analyzed using the LuminexH 100TM System (Luminex Corp.; Austin, TX) and quantified using MiraiBio MasterPlexH CT and QT Software (Hitachi Software Engineering America Ltd.; San Francisco, CA), and concentrations were expressed in pg/mL unless otherwise noted. The cytokines analyzed were IL-1α, IL-1β, IL-2, IL-3, IL-4, IL-5, IL-6, IL-9, IL-10, IL-12(p40), IL-12(p70), IL-13, IL-17, eotaxin, G-CSF, GM-CSF, IFN-γ, KC, MCP-1, MIP-1α, MIP-1β, RANTES, and TNF-α with Bio-Plex Pro^™^ Mouse Cytokine Grp I Panel 23-Plex as previously studied [8] and IL-15 and IL-18 were measured using Bio-Plex Pro^™^ Mouse Cytokine Grp II Panel 9-Plex (Bio-Rad; Hercules, CA).

### Measurements of Corticosterone, CRP, C3, and Flt-3 ligand

Corticosterone (Abcam, Cambridge, MA), CRP, C3 (GenWay, SanDiego, CA), and Flt-3 ligand (R & D System, Minneapolis, MN) were measured with commercial ELISA kits following the manufacturer’s protocol and reported with a unit of pg/ml [10].

### Histopathology assessment

Ileum tissue specimens were collected from the mice on day 3 and day 15 (N = 6 mice per group, radiation dose = 8.75 Gy). Specimens were rinsed in cold saline solution and immediately fixed in 10% phosphate-buffered formalin. The tissue was then embedded in paraffin, sectioned transversely and stained with Hematoxylin and Eosin (H&E). Villus height and width, crypt depth, and crypt counts were measured [8] using NanoZoomer 2.0RS (Hamamatsu Corp., Bridgewater NJ). The mucosal damage of ileum for each slide was graded on a six-tiered scale defined by Chiu et al. [36] as follows: grade 0 = normal mucosa; grade 1 = development of subepithelial spaces near the tips of the villi with capillary congestion; grade 2 = extension of the subepithelial space with moderate epithelial lifting from the lamina propria; grade 3 = significant epithelial lifting along the length of the villi with a few denuded villus tips; grade 4 = denuded villi with exposed lamina propria and dilated capillaries; and grade 5 = disintegration of the lamina propria, hemorrhage, and ulceration.

### Detection of bacteria

Treated (radiation dose = 8.75 Gy) and sham-operated mice, euthanized or recently deceased (within 2 hours), were dissected aseptically to isolate bacteria from selected tissues. Facultative bacteria were isolated from these tissues according to routine standard microbiological procedures. The apex of the heart was cut and the cut surface was immediately applied directly to 5% sheep blood agar (SBA), colistin-nalidixic acid in 5% sheep blood agar (CNA), and xylose-lysine-desoxycholate agar (XLD) medium (BD Diagnostics, Sparks, MD). Samples of liver and ileum were removed and homogenized by crushing with a sterile cotton or polyester swab in a sterile petri dish and inoculated immediately onto SBA, CNA and XLD medium with a dilution-streak method. SBA and CNA were incubated in 5% CO_2_ at 35°C for 18–24 hours, XLD plates were incubated at 35°C. SBA is an enriched, non-selective medium, whereas CNA is selective for Gram-positive bacteria and XLD is selective for Gram-negative bacteria. Cultures without bacterial growth after 24 hours were incubated for another 24 hours. Single colonies of isolated microorganisms were Gram-stained and identified by a Vitek2 Compact automated system (bioMérieux, Inc., Durham, NC).

### Activated caspase-3 measurements

Activated caspase-3 protein levels were measured using Quantikine ELISA kit according to the manufacturer’s protocol and reported with a unit of ng/ml (R&D SYSTEM, Minneapolis, MN).

### Tissue lysates

Samples collected from ileum (N = 6/group) were mixed with Na^+^ Hanks’ solution, homogenized using Bullet Blender Homogenizer Storm 24 for 4 min at speed 10 (Next Advance, Averill Park, NY), and centrifuged at 9,000 x g for 10 min (Sorvall Legend Micro 21 Centrifuge, Thermo Electron Corp, Madison, WI). Supernatant fluids were conserved for protein determination and stored at −70°C until use.

### Western blots

Total concentrations of NF-κB-p65, NF-κB-p50, iNOS, ERK, JNK, p38, and IgG were determined in ileal lysates. Total protein in the cell lysates was determined with Bio-Rad reagent (Bio-Rad; Richmond, CA). Samples with 20 μg of protein in tris buffer (pH = 6.8) containing 1% sodium dodecyl sulfate (SDS) and 1% 2-mercaptoethanol were resolved on SDS-polyacrylamide slab gels (Novex precast 4–20% gel; Invitrogen; Carlsbad, CA). After electrophoresis, proteins were blotted onto a polyvinylidene difluoride (PVDF) membrane (0.45 μm; Invitrogen), using a Trans-Blot Turbo™ Transfer System and the manufacturer's protocol (Bio-Rad, Hercules, CA). After blocking the nitrocellulose membrane by incubation in tris-buffered saline-0.5% tween20 (TBST) containing 3% nonfat dried milk for 90 min at room temperature, the blot was incubated for 60 min at room temperature with antibodies directed against NF-κB-p50, NF-kB-p65, IgG, p-ERK, p-JNK, and p-p38 (Santa Cruz Biotechnology; Santa Cruz, CA), iNOS (BD Bioscience, San Jose, CA), and STAT3 (Cell Signaling Technology, Danvers, MA) at a concentration of 1 μg/ml in TBST—3% dry milk. The blot was then washed 3 times (10 min each) with TBST before incubating the blot for 60 min at room temperature with a 1000x dilution of species-specific IgG peroxidase conjugate (Santa Cruz Biotechnology) in TBST. The blot was washed 6 times (5 min each) in TBST before detection of peroxidase activity using the Enhanced Chemiluminenscence Plus (Amersham Life Science Inc., Arlington Heights, IL, USA). IgG levels were not altered by radiation and were used as a control for protein loading. Protein bands of interest were quantitated densitometrically and normalized to IgG. Data were expressed as intensity ratio to IgG, because IgG levels present in tissues was not affected after irradiation [8].

### RNA Isolation and quantitation

Total RNA was isolated from serum and kidney samples using the miRNeasy Serum/Plasma Kit (Cat# 217184; Qiagen Inc. CA) and the mirVana miRNA isolation kit (Cat# AM1560; Life Technologies, Carlsbad, CA, USA) respectively, according to the manufacturer's protocol. Briefly, Qiazol lysis reagent (500μl) was added to the serum sample (100 μL), vortexed, and incubated at room temperature for 5 min. Chloroform (100 μL) was added to the lysate, gently mixed, and centrifuged for 15 min at 12,000×g at 4°C. The aqueous phase obtained after centrifugation was mixed with 1.5 volume of 100% ethanol and loaded on RNeasy MiniElute spin columns. The flow-through fluid, after a brief centrifugation, (8,000 x g) was discarded and the column was washed with 700 μL of Buffer RWT, 500 μL of Buffer RPE, and 500 μL of 80% ethanol. The RNA was eluted with RNase-free water. For ileal tissue, 20x volume of lysis buffer was added to the sample and homogenized on ice followed by adding 1/10^th^ volume of homogenate additive to the lysate. Samples were incubated at 4°C for 10 min and equal volume of phenol:chloroform was added to the tissue lysate, vortexed, and centrifuged for 8 min at 12,000 × g. The aqueous layer was collected after centrifugation and mixed with 1.25 volumes of absolute ethanol and passed through the RNAqueous micro kit cartridge. The flow-through fluid was discarded and the column was washed once with 700 μL of wash solution-1, twice with 500 μL of wash solution-2/3. RNA was finally eluted in pre-heated (37°C) RNase-free water.

Both quality and quantity of the miRNA in the total RNA isolated were measured and analyzed using Agilent Small RNA kit (Cat#5067–1548; Agilent Technologies, Santa Clara, CA, USA) in Agilent 2100 Bioanalyzer.

### MiRNA profiling

Complementary DNA was synthesized from total RNA, which contained 5 ng of miRNA from serum or 500ng of total RNA from ileum tissue, by reverse transcription (RT) with TaqMan miRNA RT Kit (Life Technologies, Carlsbad, CA, USA) as described before [17]. RT was performed using RNA samples with megaplex pools of stem-loop RT primers for pool A/B; and TaqMan miRNA RT kit (Applied biosystems Inc., Carlsbad, CA). Briefly, the RT reaction mixture contained 0.8 μl Megaplex RT primers Rodent Pool A/B (v3.0), 0.2 μl 100 mM dNTPs (with dTTP), 1.5 μl Multiscribe reverse transcriptase (50 U/μl), 0.8 μl 10× RT Buffer, 0.9 μl MgCl_2_ (25 mM), 0.1 μl RNAse inhibitor (20 U/μl), RNA template and nuclease-free water to a final volume of 7.5 μl. RT reaction was carried out on Veriti 96-Well Thermal Cycler (Life Technologies, Carlsbad, CA, USA) with the following reaction conditions: [16°C/2min; 42°C/1min; 50°C/1sec] X 40 cycles; 85°C/5 min; and hold at 4°C. Pre-amplification of serum RT products was performed using the pre-amplification master mix and primer set (Life Technologies, Carlsbad, CA, USA) according to the manufacturer’s protocol with the following reaction conditions: 95°C/10min; 55°C/2min; 72°C/2min; [95°C/15sec; 60°C/4min] X 16 cycles; 99°C/10min; and held at 4°C. The undiluted pre-amplification products from serum samples or undiluted RT product from ileum samples were used for the miRNA profiling using TaqMan Low-Density Rodent microRNAs Array (TLDA) Set v3.0 (Applied Biosystems, Life Technologies, Foster City, CA) containing 561 rodent miRNAs according to the manufacturer’s protocol. The quantitative RT-PCR (qRT-PCR) reaction was carried out at default thermal-cycling conditions in ABI 7900HT Fast Real-Time PCR System (Applied Biosystems).

### Data analysis of miRNA array

Real-time PCR raw data was analyzed using the Real-Time StatMiner^®^ Software V.4.5.0.7 (Integromics, Madison, WI). The data was normalized to U6 snRNA as an optimal endogenous control. Relative quantitation (RQ) of miRNA expression between control and injured groups was done by filtering of miRNAs having expression control (Ct) values below 35 cycles and the detection of expression in all biological replicates of calibrator and target. The miRNAs that had more than a two-fold modulation with a P value <0.05 were considered as significantly modulated miRNAs. Both functional and network analysis of altered miRNA and their gene targets were performed using the Ingenuity Pathway Analysis (IPA) program (Ingenuity Systems Inc, Redwood City, CA).

### Statistical analysis

All results are expressed as means ± SEM. One-way ANOVA, two-way ANOVA, Bonferroni’s inequality, studentized-range test, *χ*^2^ test, and Student’s *t*-test were used for comparison of groups and paired samples as appropriate. For all data, statistical significance was accepted at p<0.05.

## Results

### Hemorrhage did not alter radiation-induced corticosterone stress responses

**Fig 1:**
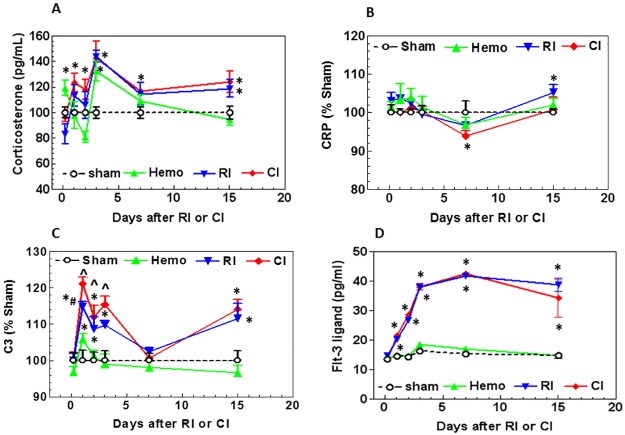
Hemorrhage enhances the CRP and C3 responses and the early corticosterone response to radiation in the serum, but not the late corticosterone or Flt-3 ligand response. Mice received 8.75 Gy γ-photon radiation. Blood was collected at several time points after RI and CI from the surviving mice (N = 6 per group at each time point). Corticosterone (A), CRP (B), C3 (C), and Flt-3 ligand (D) in serum were measured. All values are means±SEM. *p<0.05 vs. sham; ^p<0.05 vs. Sham: free of any injury; RI. Hemo: hemorrhage; RI: radiation injury; CI: RI+Hemo.

Corticosterone is the main glucocorticoid involved in regulation of stress responses. Our laboratory previously reported that RI after exposure to neutron/γ-ray mixed field increased corticosterone levels [10]. To determine whether RI, Hemo, or CI in the present model generated the same stress level or not, corticosterone concentrations in plasma were measured at multiple time points. Fig 1A shows that Hemo induced a significant increase in corticosterone concentrations 4 hours post-Hemo whereas RI decreased the concentration and CI did not alter corticosterone compared to the sham concentrations. On day 1, the corticosterone concentration in Hemo mice returned to the baseline but corticosterone concentration in RI and CI mice began to increase. On day 2, the corticosterone concentrations in Hemo mice continued to decrease below the baseline but the corticosterone concentrations in RI and CI mice remained elevated. On day 3, the corticosterone increases peaked with RI, CI and post-Hemo. The elevation in the Hemo mice returned to the baseline on days 7 and 15. In contrast, the corticosterone concentrations remained significantly elevated above the basal levels until day 15 in RI and CI mice. There were no significant differences between the elevations of coticosterone with RI and CI during the evaluation period.

### Hemorrhage potentiates radiation-induced systemic bacterial infection

Considering that CI promoted greater systemic bacterial infection after ionizing irradiation alone [8], bacteria were cultured from circulating heart blood, liver, and spleen of sham, Hemo, RI, and CI mice on days 7 and 15. On day 7, no bacteria were detected in all mice that were evaluated (N = 6 per group). On day 15, *Staphylococcus warneri* (Gram-positive) was detected in only 1 sham mouse (N = 6). No bacteria was identified in Hemo mice (N = 6). However, *Proteus mirabilis* (Gram–negative) was found in 1 of 6 surviving RI mice (N = 6), whereas *Streptococcus sanguinis* (Gram-positive) and *Sphingomonas paucimobilis* (Gram-negative) were detected in 2 of 3 surviving CI mice (N = 6 initially; three mice were diseased between day 7 and day 15 due to inflammation and infection).

### Hemorrhage diminishes the CRP response to radiation

Because radiation increases CRP in anemic cancer patients [37] and irradiated mice [10], CRP in the serum was measured in Hemo, RI, and CI mice. Fig 1B shows that CRP did not significantly increase in Hemo mice during the 15-day experimental period. The CRP increased slightly and transiently in RI mice on day 1, decreased slightly by day 7, and rose again on day 15. CRP in CI mice decreased below the baseline on day 7 but returned to the baseline on day 15.

### Hemorrhage enhances the early C3 response to radiation injury

C3 plays a central role in the activation of the complement system [28]. Its activation is required for both classical and alternative complement activation pathways. Persons who have a C3 deficiency are susceptible to bacterial infections [28]. Moreover, as discussed above, traumatic CI resulted in an early onset of bacterial infection and the occurrence of sepsis, a severe systemic bacterial infection [8]. Both wound and burn trauma enhanced radiation-induced increases in C3 [10]. Therefore, C3 was measured in the mice of the present experiment.

Fig 1C shows that the C3 concentration in Hemo mice increased significantly by 5.83±1.45% (p<0.05) above the sham control on day 1 post-Hemo, then returned to the baseline level on day 2, and remained at the baseline level up to day 15. C3 in RI mice increased significantly by 14.81±1.36% (p<0.05) above the sham control on day 1 post-RI. The increase in concentration returned to the baseline level by day 7 but became significantly elevated again by day 15. C3 in CI mice increased still further by 21.08±1.79% (p<0.05) on day 1 post-CI, remained high on day 2 and day 3, and then returned to the baseline on day 7, but significantly increased again on day 15 (p<0.05) similar to the RI level, confirming the presence of sepsis caused by RI and CI on day 15.

### Hemorrhage did not alter flt-3 ligand responses to radiation in CI vs. RI

Flt-3 ligand is a bio-indicator for bone marrow aplasia and its release may be triggered by stem cell deficiency in the bone marrow. Its concentration in serum is inversely correlated to the bone marrow integrity [38–40]. In our study, Flt-3 ligand concentration was measured in serum at several time points post-Hemo, -RI, and -CI. Fig 1D shows that the Flt-3 ligand concentration in Hemo mice remained at basal levels. Flt-3 ligand concentrations increased notably in RI mice by 40–171% (p<0.05) above the sham controls by day 7 and remained at this level up to day 15. Flt-3 ligand concentrations in CI mice were similar to those in RI mice. That is, Flt-3 ligand in RI and CI mice increased in a similar manner at each time point post-RI and -CI.

### Hemorrhage augmented early RI-induced increase in serum concentrations of cytokines and chemokines

**Fig 2:**
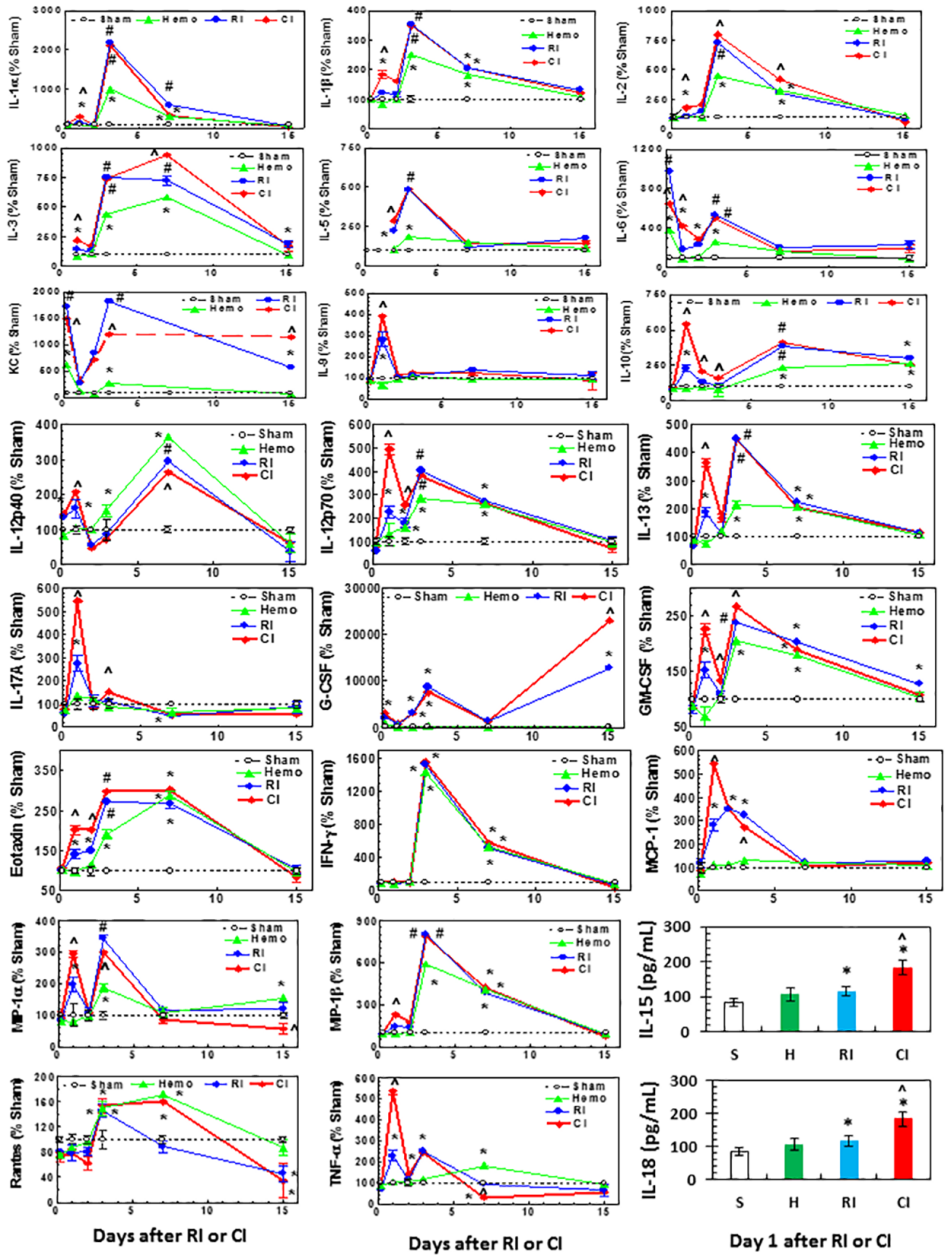
CI increases cytokines and chemokines in serum more than RI. Mice received 8.75 Gy γ-photon radiation. Blood was collected at several time points after RI (N = 6 per group at each time point). Cytokines and chemokines in serum were measured. The values were % to sham day 1. For IL-15 and IL-18, the sera on day 1 were used to measure these 2 cytokines because data with other cytokines and chemokines suggest a enhancement was present on day 1. All values are means±SEM. *p<0.05 vs. sham; ^p<0.05 vs. RI; #p<0.05 vs. sham and Hemo. Sham: free of any injury; Hemo: hemorrhage; RI: radiation injury; CI: RI+Hemo.

Our laboratory has reported that RI significantly increases IL-6, IL-10, KC, G-CSF, and MCP-1, whereas wound trauma following RI augmented increases in IL-1β, IL-6, IL-9, IL-10, IL-13, KC, G-CSF, eotaxin, INF-γ, MCP-1, and MIP-1α, and MIP-1β [8]. Therefore, in these experiments we measured numerous cytokine and chemokine concentrations in serum using Bio-Plex Pro^™^ Mouse Cytokine Grp I Panel 23-Plex. Fig 2 shows that concentrations of the following cytokines and chemokines increased on day 3, day 7, or both in Hemo mice: IL-1α, -1β, IL-2, IL-3, IL-5, IL-6, KC, IL-10, IL-12p40, IL-12p70, IL-13, GM-CSF, eotaxin, IFN-γ, MIP-1α, MIP-1β, RANTES, and TNF-α. In RI mice, concentrations of IL-1α, IL-1β, IL-2, IL-3, IL-5, IL-6, KC, IL-9, IL-10, IL-12p40, IL-12p70, IL-13, IL-17A, G-CSF, GM-CSF, eotaxin, IFN-γ, MCP-1, MIP-1α, MIP-1β, RANTES, and TNF-α. With RI the levels of these cytokines and chemokines were also increased on day 3, or 7, or both at greater levels than in Hemo alone mice except in IL-12p40, IFN-γ, and RANTES. In CI mice, concentrations of IL-1α, IL-1β, IL-2, IL-3, IL-9, IL-10, IL-12p40, IL-12p70, IL-13, IL-17A, GM-CSF, eotaxin, MCP-1, MIP-1α, MIP-1β, and TNF-α transiently increased on day 1, while concentrations of G-CSF increased remarkably higher on day 15 as well.

Because most of the CI-induced enhancement of cytokines was occurred on day 1, we measured IL-15 and IL-18 in serum on day 1 using Bio-Plex Pro^™^ Mouse Cytokine Grp II Panel 9-Plex. Hemo did not alter IL-15 and IL-18 concentrations, whereas RI significantly increased them and CI further increased them. The data are expressed in pg/mL.

### Hemorrhage augmented RI-induced increases in cytokines and chemokines concentrations in the ileum

**Fig 3:**
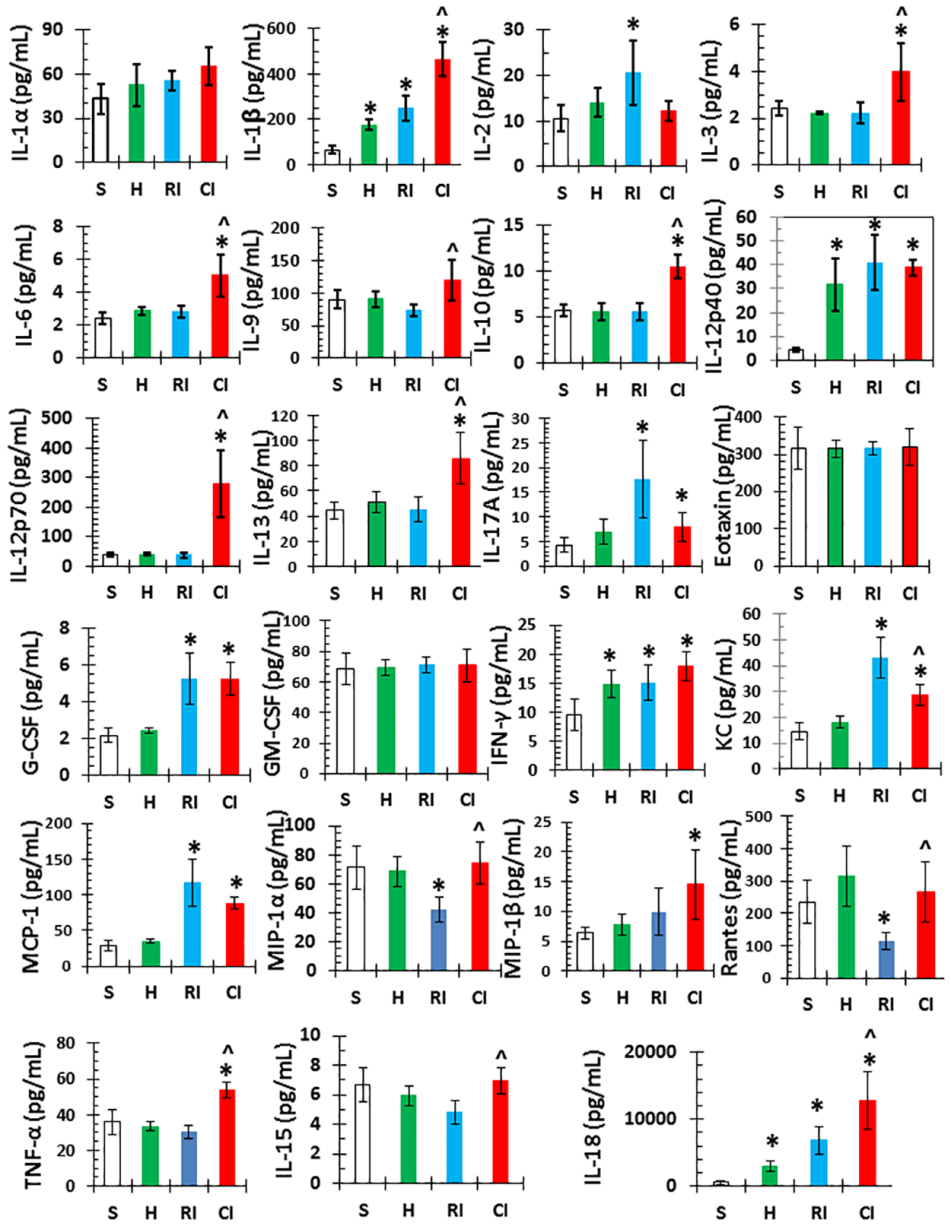
Ileal cytokines and chemokines are increase more in CI than RI. Mice received 8.75 Gy γ-photon radiation. Ileum samples were collected on day 1 after RI (N = 6 per group). Cytokines and chemokines in ileum lysates on day 1 after RI and CI were measured. All values are means±SEM. *p<0.05 vs. sham; ^p<0.05 vs. Sham: free of any injury; RI. Hemo: hemorrhage; RI: radiation injury; CI: RI+Hemo.

Since cytokines are known to be produced by macrophages, osteoblasts, smooth muscle cells, fat cells, T cells, monocytes, epithelial cells, bone marrow, ileum, spleen, and kidney [17, 41–45], and because Fig 2 shows the CI-induced cytokine/chemokine augmentation occurred on day 1, tissue lysates of ileum collected on day 1 were evaluated for the concentrations of cytokines similar to the serum evaluation. Fig 3 shows that IL-1β, IL-12p40, and IFN-γ are significantly increased in Hemo mice compared to sham; IL-1β, IL-2, IL-12p40, IL-17A, IL-18, KC, G-CSF, IFN-γ, and MCP-1 increased but IL-15, MIP-1α, and RANTES decreased in RI mice; and IL-1β, IL-3, IL-6, IL-9, IL-10, IL-12p70, IL-13, IL-18, and TNF-α were transiently augmented in CI mice. Table 1 shows the cytokine/chemokine augmentation in the ileum and serum of CI mice, suggesting that ileal cytokines may have contributed to elevations of cytokines and chemokines in serum (Table 1).

**Table 1:** Hemorrhage in the CI model magnified cytokines/chemokines in serum and ileum of CI mice.

#	Acute (1 day after irradiation)	Serum	Ileum
**1**	**IL-1α**	√	
**2**	**IL-1β**	√	√
**3**	**IL-2**	√	
**4**	**IL-3**	√	√
**5**	**IL-5**	√	
**6**	**IL-6**	√	√
**7**	**KC**	√	
**8**	**IL-9**	√	√
**9**	**IL-10**	√	√
**10**	**IL-12p40**	√	
**11**	**IL-12p70**	√	√
**12**	**IL-13**	√	√
**13**	**IL-15**	√	
**14**	**IL-17A**	√	
**15**	**IL-18**	√	√
**16**	**G-CSF**		
**17**	**GM-CSF**	√	
**18**	**Eotaxin**	√	
**19**	**IFN-γ**		
**20**	**MCP-1**	√	
**21**	**MIP-1α**	√	
**22**	**MIP-1β**	√	
**23**	**Rantes**		
**24**	**TNF-α**	√	√

√: Magnification

### Hemorrhage augments RI-induced mucosal injury in ileum

**Fig 4:**
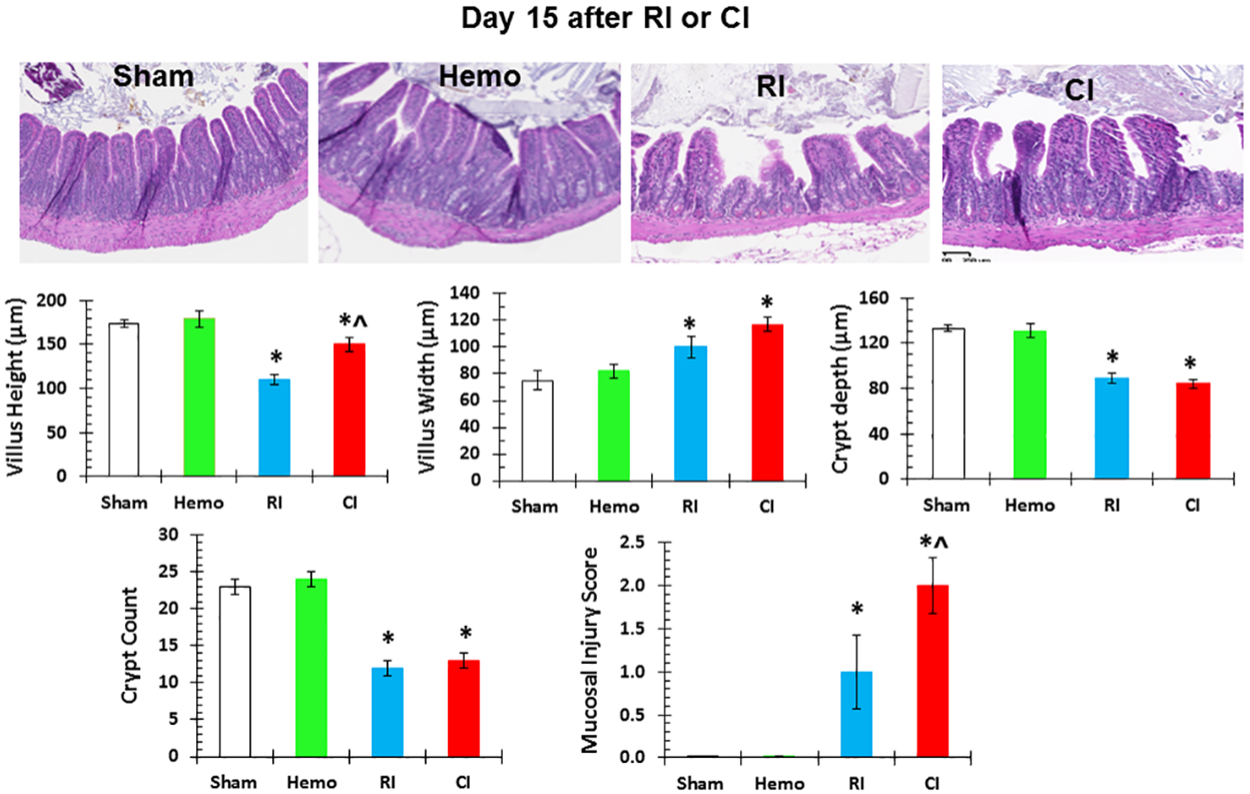
CI induces ileum damage more than RI. Mice received 8.75 Gy γ-photon radiation. Ileum samples were collected on day 15 after RI (N = 4 per group) for histology stained with H&E. All values are means±SEM. *p<0.05 vs. sham; ^p<0.05 vs. RI. Sham: free of any injury; Hemo: hemorrhage; RI: radiation injury; CI: RI+Hemo.

Because significant increases in proinflammatory cytokines were demonstrated in the ileum of RI mice and augmented in CI mice, the histopathology of the ileum on day 3 and day 15 was examined by measuring villus height, villus width, crypt depth, and crypt counts as well as assigning a mucosal injury score to determine if there is a relationship between the inflammatory responses and recession of villi after Hemo, RI, and CI. On day 3 and day 15, villus height, villus width, crypt depth, and crypt counts were not altered in Hemo mice where the mucosal injury score was 0. On day 3, villus height, villus width, crypt depth, and crypt counts were not altered in RI mice and CI mice where the mucosal injury score was 0 as well (data not shown). On day 15, villus height, crypt depth, and crypt counts were decreased, but villus width increased and the mucosal injury score was approximately 1 in RI mice. However, villus width was greater and mucosal injury scores were elevated in CI mice even though CI attenuated the RI-induced shortening of villi on day 15, compared to those in RI mice on day 15. Fig 4 shows that CI-induced ileum damage more than RI on day 15.

### Hemorrhage augments RI-induced apoptosis in ileum

**Fig 5:**
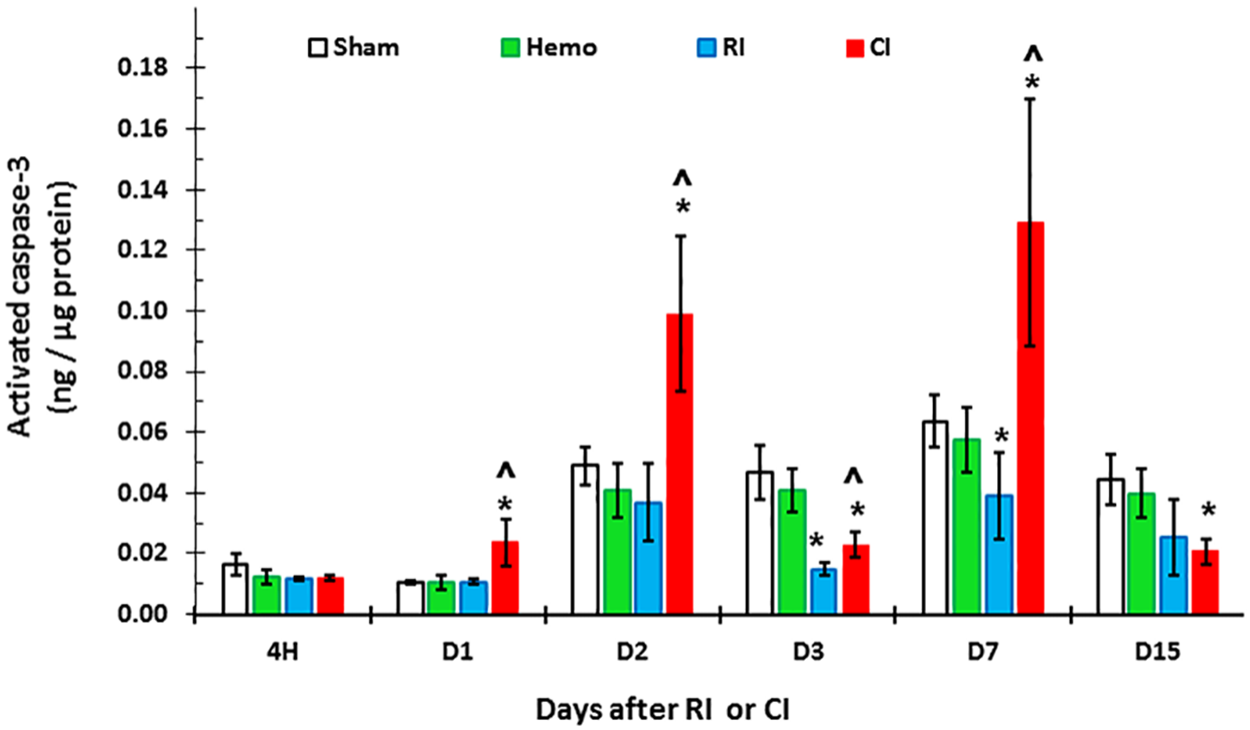
CI increases activated caspase-3 in ileal mucosa more than RI. Mice received 8.75 Gy γ-photon radiation. Ileum samples were collected at several time points after RI (N = 6 per group at each time point). Activated caspase-3 concentrations in ileum lysates were measured. All values are means±SEM. *p<0.05 vs. sham; ^p<0.05 vs. RI. Sham: free of any injury; Hemo: hemorrhage; RI: radiation injury; CI: RI+Hemo.

Since CI augmented proinflammatory cytokine concentrations in serum and ileum, and because edema was noted in villi (width of the villi), caspase-3 activation was measured to determine if apoptosis occurred in ileum. Fig 5 shows that activated caspase-3 concentration progressively increased in CI-mouse ileum beginning on day 1 through day 7 (except day 3), but decreased on day 15.

### Hemorrhage in CI augments RI-induced NF-κB, STAT3 and iNOS in ileum

**Fig 6:**
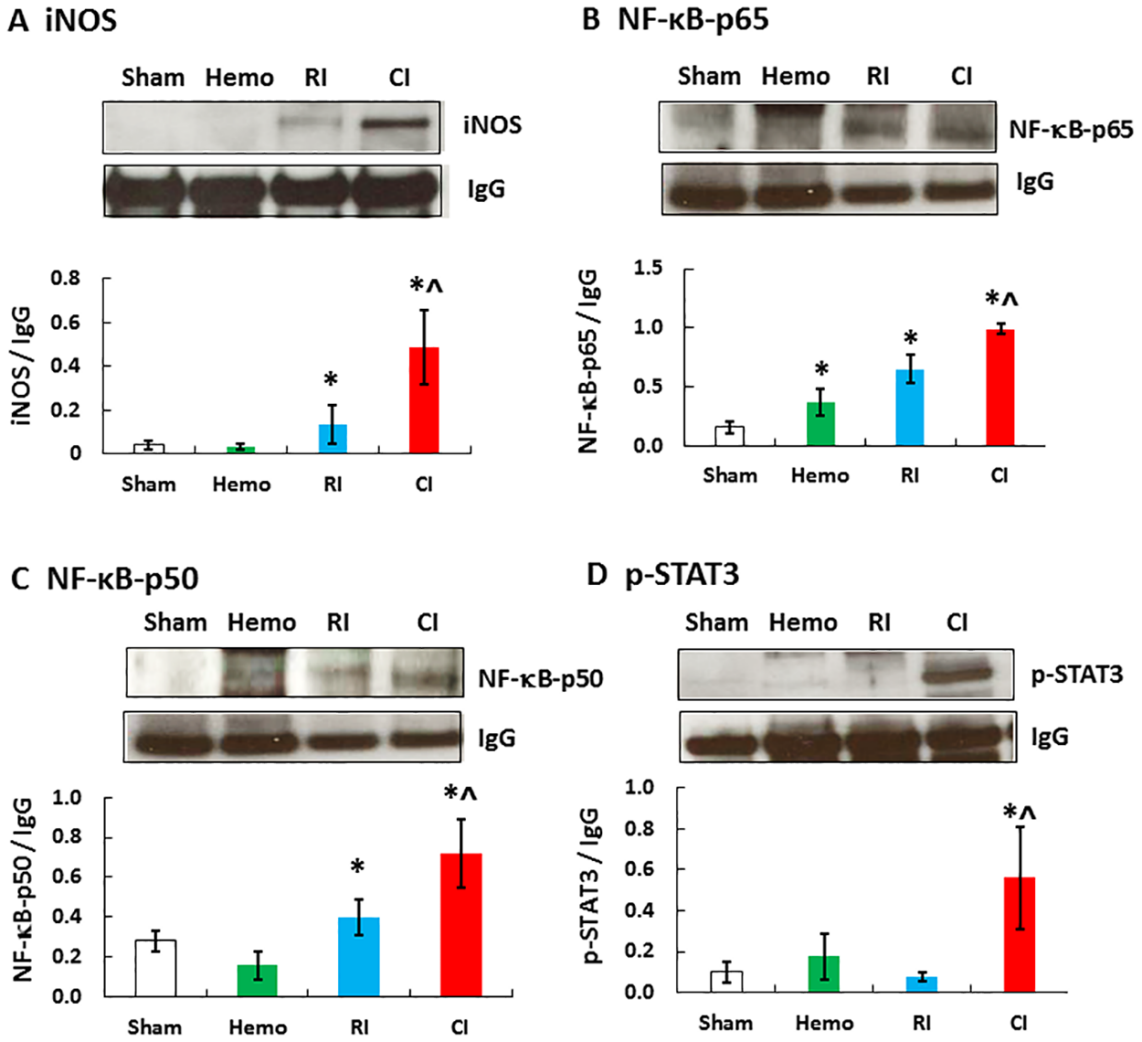
CI increases iNOS, NF-κB, and STAT3 in ileum more than RI. Mice received 8.75 Gy γ-photon radiation. Ileum lysates 1 day after RI were immunoblotted to detect iNOS, NF-κB p-65, p-50, and phosphorylated STAT3 (N = 4 per group). All values are means±SEM. *p<0.05 vs. sham; ^p<0.05 vs. RI. Sham: free of any injury; Hemo: hemorrhage; RI: radiation injury; CI: RI+Hemo.

Increased concentration of activated caspase-3, which we detected and show in Fig 5, would depend upon increased iNOS expression [8] in the ileum which leads to apoptosis [46]. It is known that NF-κB and STAT3 regulate iNOS gene expression [47]. Therefore, NF-κB, STAT3, and iNOS protein amounts in the ileum were measured using Western blot analysis. Fig 6 shows that NF-κB, STAT3, and iNOS protein amounts in ileum were significantly increased in the ileum of CI mice on day 1 over RI or Hemo alone.

### Hemorrhage augments RI-induced phosphorylation of ERK and p38 in ileum

**Fig 7:**
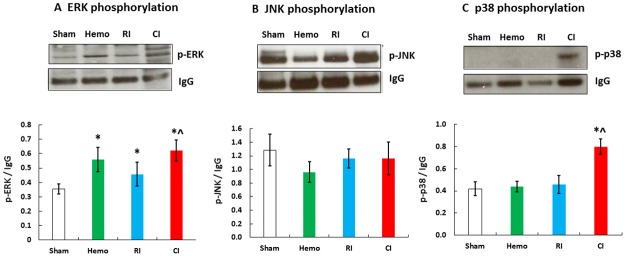
CI increases MAPK activation in ileum more than RI. Mice received 8.75 Gy γ-photon radiation. Ileum lysates 1 day after RI were immunoblotted to detect ERK, JNK, and p38 (N = 4 per group). All values are means±SEM. *p<0.05 vs. sham; ^p<0.05 vs. RI. Sham: free of any injury; Hemo: hemorrhage; RI: radiation injury; CI: RI+Hemo.

MAPK is known to promote NF-κB [48]. Therefore, ERK, JNK, and p38 phosphorylation were evaluated. As shown in Fig 7, ERK phosphorylation was significantly increased in ileum of Hemo-only and RI-only mice, whereas ERK phosphorylation increased further in CI mice. Phosphorylation of p38 was significantly increased in ileum of CI mice but not in Hemo or RI mice. JNK phosphorylation was not significantly altered in ileum by Hemo, RI, or CI.

### Hemorrhage alters RI-induced miRNA expression in the serum and ileum and their association with inflammation

We observed that activation of caspase-3 increased in ileum of CI mice and the levels of numerous cytokines/chemokines were elevated in serum and ileum on day 1. Thus, we evaluated the effect of Hemo, RI, and CI on the expression of miRNAs levels, and the target of those molecules in the serum and ileum. The complete miRNA-expression profile in the serum on day 1 after CI identified 18 miRNAs, which were differentially expressed in comparison to the control. Among them, 8 and 10 miRNAs were up- and down- regulated, respectively (Table 2).

**Table 2:** Alteration of specific miRNAs in serum of CI mice.

S#	Name of miRNA	Fold expression	P value
**1**	**mmu-miR-125a-3p**	491.83	0.003
**2**	**mmu-miR-434-3p**	13.77	0.025
**3**	**mmu-miR-125b-5p**	6.69	0.038
**4**	**mmu-miR-342-3p**	5.53	0.007
**5**	**mmu-miR-34b-3p**	5.16	0.028
**6**	**mmu-miR-877-3p**	3.23	0.034
**7**	**mmu-miR-463-5p**	3.06	0.050
**8**	**mmu-miR-503-3p**	2.83	0.042
**9**	**mmu-miR-29c**	-3.02	0.045
**10**	**mmu-miR-15a**	-3.09	0.036
**11**	**rno-miR-148b-5p**	-3.84	0.040
**12**	**mmu-miR-195**	-3.98	0.027
**13**	**mmu-miR-301a**	-4.35	0.035
**14**	**mmu-miR-362-3p**	-6.70	0.010
**15**	**mmu-miR-701**	-8.61	0.006
**16**	**mmu-miR-669a**	-743.53	0.018
**17**	**rno-miR-196c**	-1374.77	0.024
**18**	**mmu-miR-363**	-2184.11	0.039

Meanwhile, the miRNA-expression profiles in the ileum of CI mice identified 22 miRNAs, which were differentially expressed on day 1 after CI. Among them, 16 and 6 miRNAs were up- and down- regulated, respectively (Table 3).

**Table 3:** Alteration of specific miRNAs in ileum of CI mice.

S#	Name of miRNA	Fold expression	P Value
1	**mmu-miR-34a**	37.49	0.031
2	**mmu-miR-148b**	30.63	0.022
3	**mmu-miR-129-3p**	17.13	0.039
4	**hsa-miR-15b**	5.23	0.045
5	**mmu-miR-188-5p**	3.83	0.019
6	**mmu-miR-99a**	3.65	0.011
7	**mmu-miR-26b**	3.52	0.025
8	**mmu-miR-32**	3.15	0.012
9	**mmu-miR-410**	3.14	0.014
10	**mmu-miR-465b-5p**	3.10	0.039
11	**mmu-miR-100**	2.89	0.026
12	**mmu-miR-26a**	2.56	0.033
13	**mmu-miR-126-5p**	2.47	0.034
14	**mmu-miR-200b**	2.34	0.030
15	**mmu-miR-98**	2.20	0.016
16	**mmu-let-7g**	2.03	0.032
17	**hsa-miR-151-5p**	-2.11	0.045
18	**mmu-miR-1839-3p**	-2.49	0.013
19	**mmu-miR-1839-5p**	-2.57	0.008
20	**rno-miR-20b**	-3.19	0.037
21	**rno-miR-148b-5p**	-10.38	0.019
22	**mmu-miR-495**	-33.23	0.009

**Fig 8:**
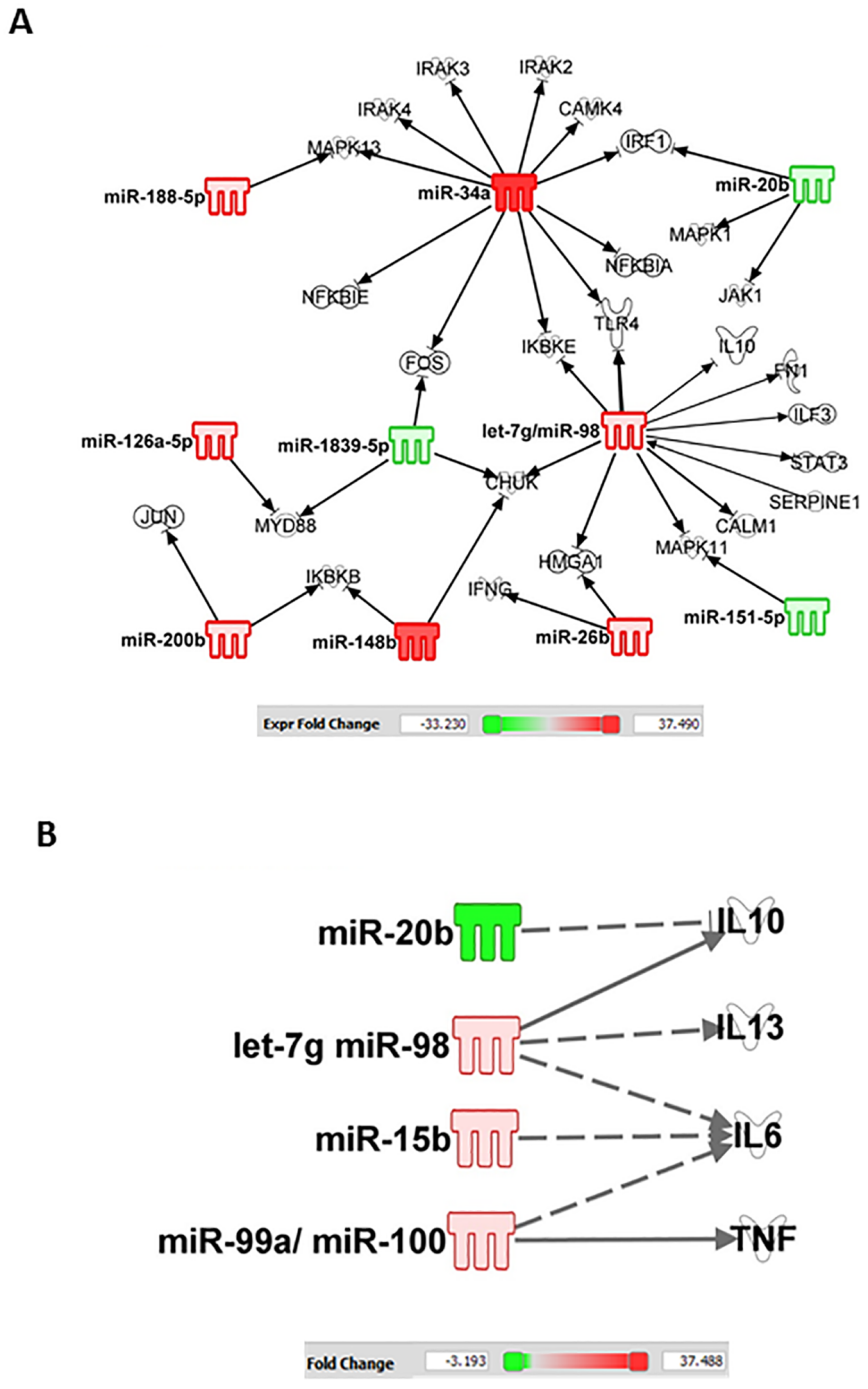
CI alters the miRNAs in ileum. Ingenuity Pathway Analysis filtered experimentally validated and direct gene targets of (A) NF-κB, MAPK, and STAT3, and (B) cytokines/chemokine in ileum of CI mice.

Analysis of these miRNAs for their mRNA targets, with particular reference to bacterial infection and inflammatory cytokine/chemokine responses in the ileum was carried out using the IPA program. In the ileum, IPA indicated that increases in miR-34a-5p alters NF-κB; let-7g and miR-98 regulates STAT3; miR-34a, mR-188-5p, let-7a-5p, and miR-151-5p regulate MAPK; miR-20b regulates IL-10; let-7g and miR-98 regulate IL-10, IL-13, IL-6; miR-15b regulates IL-6; whereas miR-99a and miR-100 regulate TNF (Fig 8).

**Fig 9:**
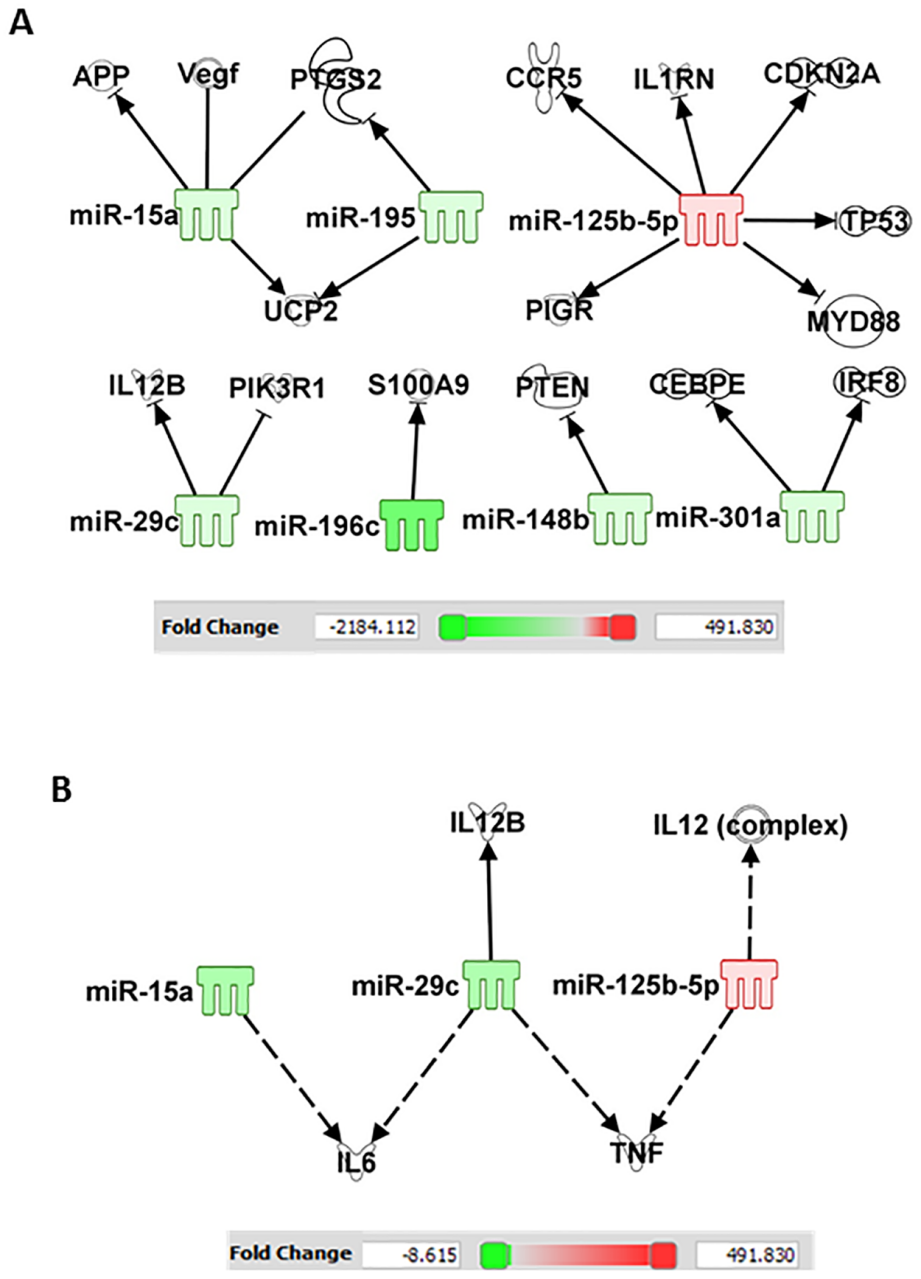
CI alters the miRNAs in serum. Ingenuity Pathway Analysis filtered experimentally validated and direct gene targets of cytokines/chemokine in serum after CI.

In the serum, IPA indicates that miR-125b alters p53, IL-12, and TNF; miR-29c alters IL-12 and IL-6; miR-15a alters VEGF and IL-6; and miR-148b alters PTEN (Fig 9).

We also found 19 miRNAs in the serum and 5 miRNAs in the ileum that were significantly altered after Hemo, RI, and CI. Table 4 shows that in the serum 5 miRNAs were upregulated and 14 miRNAs were downregulated. The data indicate the presence of the CI-induced miRNA enhancement in CI mice compared to those observed in Hemo alone or RI alone mice.

**Table 4:** Alteration of commonly present miRNAs in serum after Hemo, RI, or CI. Hemo: hemorrhage; RI: 8.75 Gy; CI: 8.75 Gy+hemorrhage.

Name of miRNA	Hemo		RI		CI	
	Fold expression	p-value	Fold expression	p-value	Fold expression	p-value
**rno-miR-224**	15449.81	9.46E-06	20110.45	6.74E-06	31406.34	2.07E-05
**mmu-miR-224**	145.6465	0.028196	76.06481	0.046283	182.2346	0.024449
**mmu-miR-685**	4.027781	0.040522	4.285297	0.035026	11.19547	0.004807
**mmu-miR-145**	5.698136	0.03228	4.089767	0.035732	12.62693	0.003343
**mmu-miR-574-3p**	6.533681	0.0207	2.738364	0.044832	12.80211	0.00086
**mmu-miR-449a**	-2.93003	0.026196	-2.66129	0.035708	-3.00685	0.030851
**mmu-miR-101a**	-3.37483	0.026736	-2.92421	0.046649	-3.91019	0.020311
**mmu-miR-103**	-2.732	0.028123	-3.02153	0.034495	-5.32129	0.00758
**mmu-miR-340-5p**	-4.07594	0.033084	-3.462	0.048917	-5.01069	0.025406
**mmu-miR-26b**	-6.59855	0.011246	-3.54044	0.034556	-19.4278	0.001208
**mmu-miR-194**	-4.18419	0.035017	-4.87095	0.021394	-13.6022	0.007058
**mmu-miR-26a**	-4.97501	0.008192	-5.85583	0.003625	-16.801	0.00037
**mmu-miR-31**	-13.0711	0.006561	-6.61528	0.018432	-28.1233	0.001128
**mmu-miR-15b**	-9.26965	0.010839	-9.18995	0.007952	-13.5844	0.004011
**mmu-miR-142-3p**	-8.14546	0.024568	-10.171	0.012167	-28.84	0.002308
**mmu-miR-24-2-5p**	-9509.2	0.002134	-2315.09	0.003565	-36391.8	2.91E-07
**mmu-let-7g-3p**	-822.116	0.000947	-5593.55	4.17E-07	-1784.82	1.93E-06
**mmu-miR-22-5p**	-5107.1	0.002919	-9612.96	0.001898	-3067.36	0.003522
**mmu-miR-322**	-5853.62	8.4E-06	-11018.1	4.03E-06	-3515.74	1.07E-05

Table 5 shows that in ileum 1 miRNA was upregulated and 4 miRNAs were downregulated. No clear patterns of miRNA regulation changes were found after Hemo, RI, and CI

**Table 5:** Alteration of commonly present miRNAs in ileum after Hemo, RI, and CI. Hemo: hemorrhage; RI: 8.75 Gy; CI: 8.75 Gy+hemorrha.

Name of miRNA	Hemo		RI		CI	
	Fold expression	p-value	Fold expression	p-value	Fold expression	p-value
**mmu-miR-1**	24.14374	0.001091	3.107536	0.008587	3.784054	0.004363
**mmu-miR-186**	7.092997	0.000227	-3.68412	0.006169	-2.059	0.022994
**hsa-miR-140**	2.111839	0.04139	-3.57629	0.002529	-3.05719	0.011996
**mmu-miR-1968**	-126.771	0.000217	-150.504	5.93E-05	-26.0804	0.015602
**mmu-miR-31**	4.46253	0.002556	-1301.46	0.003726	-811.33	0.005119

## Discussion

The condition identified as combined injury (CI) was initially described more than nine decades ago. Attention has focused on establishing useful animal-model system for evaluating the consequences of exposure to radiation in conjunction with injuries that may be associated with nuclear weapon detonation [49]. In such devastating situations, casualties are expected to overwhelm health-care facilities and, thus it is imperative to determine (1) the physiologic changes, which result from RI, tissue injury or Hemo, and their combined injury that lead to morbidity and mortality and (2) design appropriate countermeasures and interventions, which are useful for mass-casualty applications. In this study we used gamma radiation combined with Hemo as our CI, which perturbs the physiological responses of experimental animals in the serum and the ileum, an organ that is very sensitive to ionizing radiation. We showed that Hemo alone acutely elevated corticosterone, which rapidly returned to the baseline level. However, RI and CI showed similar concentrations of elevated yet sustained corticosterone in the serum (Fig 1A). These results are consistent with those of animals which received mixed-field radiation [10].

Wound [8] or skin burns [17] enhanced RI-induced increases in IL-6 concentrations in the serum. IL-6 induces the production of neutrophils [50], inhibits TNF-α and IL-1, activates IL-10, and activates transcription 3 [51, 52]. Kiang et al. [8, 53] reported that wound trauma augmented γ-radiation-induced increases in IL-6 concentrations in blood and also induced a systemic bacterial infection. An increased IL-6 concentration, in turn, upregulates expression of NF-kB and NF-IL6, which is a transcription factor that binds to the promoter region of the iNOS gene [8]. On the other hand, wound-trauma-enhanced systemic bacterial infection [8, 12, 53, 54] activates TLR-4, which increases NF-kB expression to transcribe the IL-6 gene and the iNOS gene, thus forming a positive feedback among iNOS, IL-6, NF-kB, and NF-IL6 [8], which leads to the augmented responses.

Although the mice did not reveal CRP concentrations that correlated with radiation dose or time after exposure in the literature [55], our data indicated that a transient decrease in serum CRP concentrations occurred on day 7 after CI, whereas RI alone increased serum CRP concentration on day 15, suggesting CI enhances RI-induced changes in CRP. Our data are not consistent with other reports [55]. The discrepancy could be due to the different strains of mice studied in these experiments (BALB/c vs. CD2F1). However, the time of the transiently RI-decreased CRP concentration is consistent with the observation in the scenario of radiation combined with skin burns regardless of gender [10]. CRP is recognized as a biomarker of inflammation and it is induced by a rise in IL-6, a pro-inflammatory mediator [56, 57]. But in the presence of hemorrhage, we show that reduction in CRP after radiation is further lowered by CI. On the other hand, CRP is important to promote the binding of complement components to microorganisms, and it enhances phagocytosis by CRP receptor-positive macrophages [56]. The decreases in CRP is ordinarily a self-defense response and would appear to be beneficial and desirable under these circumstances.

CI potentiated the increase in C3 on day 1, day 2, and day 3, but the increase disappeared on day 7 and then reappeared on day 15. At this time point of day 15, the C3 increases were similar between CI mice and RI mice; the CI-induced C3 enhancement was no longer present. C3 plays a central role in the activation of the complement system [28]. People with C3 deficiency are susceptible to bacterial infections [28, 58]. CI-induced C3 enhancements at early time points could be expected to protect against early bacterial infection caused by RI or CI. But that does not seem to occur. The later increases in C3 in RI mice and CI mice on day 15 (Fig 5) could be a body response targeting the CI-induced systemic bacterial infection. This observation is similar to findings after irradiation combined with wound or burns [10]. The increases in C3 are ordinarily self-defense responses and would appear to be beneficial and desirable after RI and CI. The results also confirm the presence of severe sepsis occurred after CI.

Increase in Flt-3 ligand concentration in serum after RI was not altered by Hemo. Similar results are obtained after RI combined with wounds [8]. The results suggest that Flt-3 ligand is probably a good biomarker to assess radiation doses after nuclear weapon detonation or with radiological accidents, because changes in its concentrations are specific to radiation.

Hemo augmented RI-induced increases in cytokines and chemokines in serum and ileum tissue. These results are consistent with observations in wounded mice following irradiation [8]. The ileum may have significantly contributed to increases in cytokines/chemokines in the serum. Whether other organs also contribute to these increases in serum needs to be further studied. Hemo increased RI-induced systemic bacterial infection in mice is similar to the incidence of sepsis in wounded mice following RI [8]. Whether the augmented increases in proinflammatory cytokines and chemokines such as the IL-1 family (IL-1β, IL-12p70, and IL-18), IL-6, MIP-1α, RANTES, and TNF-α in serum lead to delayed effects and late effects in tissues, including heart, lungs, kidneys, and brain, remains to be determined.

Whereas the Hemo-induced augmentation is transient, the wound-induced augmentation in a CI model is long-lasting [8]. This difference could be due to (1) difference in mouse strains, (2) difference in mouse genders, and (3) wound injury being more potent than Hemo, however, the CI-induced augmentation of cytokines/chemokines in serum and blood (Figs 2 and 3) is confirmed.

The GI tract is composed of proliferative enterocytes that are sensitive to radiation exposure. Radiation causes GI injury, including decreased villus height and increased villus width. Skin wounding following irradiation, i.e., CI, augmented the RI-induced GI mucosal injury [8]. Likewise, hemorrhagic CI in the present study induced further increases in mucosal injury and decreases in crypt depth. The augmented GI damage appeared on day 15 but not day 3 probably contributes to the increased sepsis that is observed on day 15. The association between these two outcomes requires additional investigation to elucidate the elusive mechanism.

iNOS is known to be transcribed by NF-κB and STAT3 and leads to the intrinsic apoptotic pathway [8, 46]. RI increased iNOS and CI further increased iNOS expression. These results are in agreement with those observed after CI with RI combined with wound trauma [8]. The sequential increases in iNOS protein (Fig 6) and then caspase-3 activation (Fig 5), thereby, lead to the GI mucosal injury. Furthermore, in CI mice, there is increased MAPK activity, known to upregulate NF-κB [48]. The results, taken together with iNOS increases, suggest that MAPK activation and iNOS signaling may be associated and, so, contribute to subsequent mortality.

Alterations of miRNA regulate protein translation [17, 59]. We found 18 miRNAs in serum (8 increases and 10 decreases) and 22 miRNAs in ileum (16 increases and 6 decreases) specifically modified by CI. In the ileum, using IPA, we found miR-34a was related to iNOS expression mediated by NF-κB. The promoter region of the iNOS gene contains various motifs for different transcription factors, including 10 NF-κB binding sites and 3 STAT3 sites [47]. It is reported that knockout miR-34a reduced NF-κB and iNOS expression as well as cell death [60], which supports our findings.

**Fig 10:**
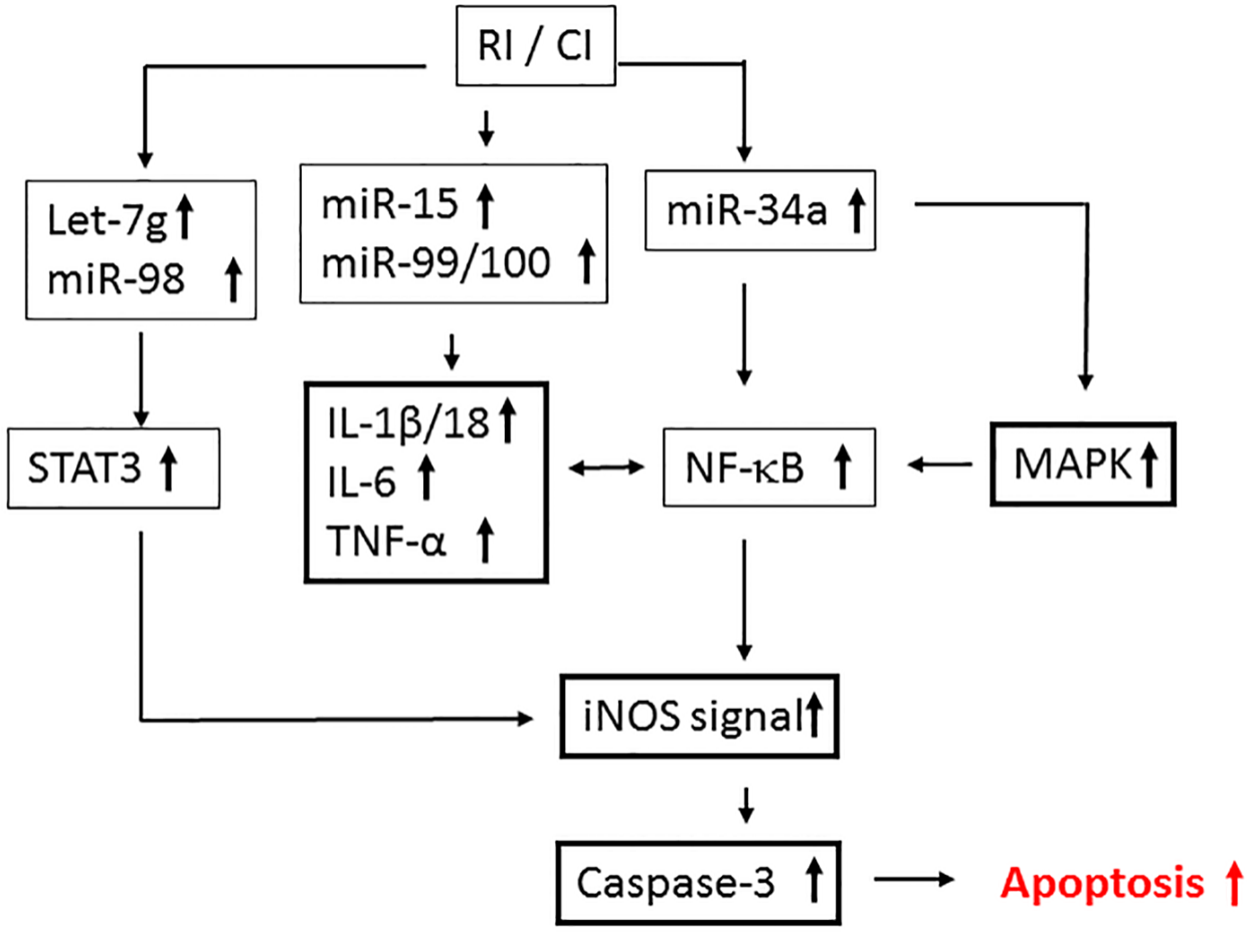
Potential scheme with possible intervention points. RI and CI increase miR-34a, which regulates MAPK and NF-κB, and let-7g and miR-98, which regulate STAT3, which, in turn, transcribes the iNOS gene. Increases in caspase-3 activation result in subsequent apoptosis [8]. RI and CI also increase miR-15, miR-99, and miR-100, which target IL-6 and TNF.

Fig 10 illustrates a potential scheme of miRNA and cellular events that may be possible intervention points based on our previously published data (references 8, 17, and 59) and the data presented in this manuscript (Figs 2, 3, 5, 6 and 7), as well as the information obtained using IPA (Figs 8 and 9). RI and CI increase miR-34a which regulates MAPK and NF-κB. MAPK activates NF-κB that transcribes the iNOS gene. As a result, increases in iNOS protein lead to caspase-3 activation and subsequent apoptosis [8]. CI increases not only miR-15, miR-99, and miR-100 that target IL-6 and TNF, but also let-7g and miR-98 that target STAT3, whose activation transcribes iNOS [61–64]. The illustration provides insight and perspective for further studies and future validation.

In summary, non-lethal Hemo after irradiation augmented RI-induced increases in C3 and decreases in CRP, but not corticosterone and Flt-3 ligand in the serum. Hemo however augmented RI-induced systemic bacterial infection in CI. Furthermore, Hemo augmented RI-induced increases in IL-1α and β, IL-2, IL-3, IL-6, IL-9, IL-10, IL-12p40 and p70, IL-13, IL-15, IL-17A, IL-18, G-CSF, GM-CSF, eotaxin, MCP-1, MIP-1 α and β, and TNF- α in serum. In the ileum, Hemo augmented IL-1β, IL-3, IL-6, IL-9, IL-10, IL-12p70, IL-13, IL-18, and TNF-α. Hemo enhanced RI-induced villus edema, decreased crypt height, and increased mucosal injury due to increased caspase-3 activation, which was mediated by increasing MAPK activation and NF-κB/iNOS signaling. Hemo increased miR34a-5p, which regulates MAPK activation and the NF-κB expression in the ileum. Also, the former was predicted to be indirectly targeted by increases in miR-188-5p, let-7, and miR-151-5p. Increases in miR-98, let-7g, miR-15b, miR-99a, and miR-100 predict to regulate STAT3, IL-10, IL-13, IL-6, and TNF. These results suggest that CI-induced alterations of these cytokines/chemokines, CRP, and C3 cause a homeostatic imbalance and may contribute to the extent of the GI injury. Inhibition of these responses may prove to be mitigative/therapeutic for CI and decrease the extent of the GI injury.
